# Spatial characterization of backpropagating action potential-evoked Ca^2+^ signals in human cortical layer 2/3 pyramidal neurons

**DOI:** 10.3389/fnsyn.2026.1769881

**Published:** 2026-02-10

**Authors:** Ildikó Szöts, Martin Tóth, Csongor Ludányi, Pál Barzó, Éva Adrienn Csajbók, Gábor Tamás, Gábor Molnár

**Affiliations:** 1 HUN-REN-SZTE Research Group for Cortical Microcircuits, Department of Physiology, Anatomy and Neuroscience, University of Szeged, Szeged, Hungary; 2 Department of Neurosurgery, University of Szeged, Szeged, Hungary

**Keywords:** backpropagating action potential, dendritic calcium signal, human neocortex, pyramidal neuron, voltage-gated calcium channels

## Abstract

**Introduction:**

In pyramidal neurons, backpropagating action potentials (bAPs) activate voltage-gated calcium channels (VGCCs), producing compartment-specific dendritic Ca^2+^ transients. While extensively characterized in rodent models, little is known about the spatial properties and channel-specific contributions of bAP-induced Ca^2+^ signals in human cortical neurons.

**Methods:**

We used simultaneous whole-cell patch-clamp recordings and two-photon Ca^2+^ imaging in acute human cortical slices to characterize bAP-evoked Ca^2+^ transients along the apical dendrites of layer 2/3 pyramidal neurons.

**Results:**

We found that Ca^2+^ signal amplitudes followed a non-linear spatial profile, increasing proximally and peaking between 50-100 µm from the soma before declining in more distal regions. Oblique dendrites exhibited significantly higher Ca^2+^ amplitudes compared to the primary apical branches. Morphological parameters, such as dendritic diameter, spine density, and branching, were correlated with the spatial profile of Ca^2+^ transients to the peak of the calcium signal profile. Pharmacological blockade of VGCCs revealed that major channel subtypes (L-, N-, R-, and T-type) contribute to dendritic Ca^2+^ influx, with distinct spatial effects. In particular, N-type channel blockade produced the largest attenuation in the medial dendritic segments, while T-type channel inhibition affected all regions.

**Discussion:**

These findings highlight spatial heterogeneity and channel-specific contributions to dendritic Ca^2+^ signaling in human neocortical neurons and underscore the influence of dendritic morphology on signal propagation.

## 1 Introduction

Neuronal calcium influx plays a critical role in numerous cellular functions, including synaptic plasticity, cellular excitability, dendritic integration, gene expression, developmental processes, and neurotransmitter release (Kennedy, 1989; Bliss and Collingridge, 1993; Berridge, 1998). Intracellular calcium elevations arise from both the release of calcium from internal stores and the influx through membrane-bound calcium channels, such as voltage-gated calcium channels (VGCCs).

Dendritic calcium influx through VGCC is primarily activated by synaptic input (Magee and Johnston, 1997; Larkum et al., 1999; Stuart and Häusser, 2001; Larkum and Zhu, 2002; Schaefer et al., 2003) or by backpropagating action potentials (bAP) (Jaffe et al., 1992; Miyakawa et al., 1992; Spruston et al., 1995; Colbert and Johnston, 1996). bAPs are antidromic action potentials initiated in the axonal initial segment that propagate retrogradely into the dendritic arbor and generate stereotyped spike-timed Ca^2+^ transients.

These bAP-induced Ca^2+^ signals are a central mechanism by which somatic firing is broadcast to dendrites to instruct synaptic plasticity. The amplitude and timing of bAP-evoked Ca^2+^ relative to synaptic input determine the sign and magnitude of long-term changes in synaptic strength in spike timing dependent plasticity (STDP) paradigms (Magee and Johnston, 1997; Markram et al., 1997; Koester and Sakmann, 1998; Caporale and Dan, 2008; Sjöström et al., 2008; Verhoog et al., 2013). More recent work has shown that the interaction between bAPs, clustered synaptic activity, and dendritic nonlinearities gives rise to dendritic spikes, local Ca^2+^ hotspots, and branch-specific potentiation, supporting the idea that bAP-mediated Ca^2+^ signaling links somatic spike patterns to spatially structured plasticity and storage of information in individual dendritic branches (Kampa et al., 2007; Grienberger and Konnerth, 2012; Bittner et al., 2015, 2017; Cichon and Gan, 2015).

The amplitude and velocity of bAPs can be influenced by several factors, including dendritic voltage-gated channels, synaptic activity, neuromodulation, and, notably, the morphological characteristics of the dendritic tree, such as dendritic diameter and branching pattern (McCormick, 1992; McCormick et al., 1993; Williams and Stuart, 2003; Oláh et al., 2025). By elevating intracellular Ca^2+^ levels in dendrites, bAPs are believed to play a key role in dendritic signal processing and synaptic plasticity (Magee and Johnston, 1997; Markram et al., 1997; Koester and Sakmann, 1998; Kampa et al., 2007; Sjöström et al., 2008; Landau et al., 2022).

VGCCs are classified into three main groups based on sequence homology and functional properties: Cav1 (L-type, high-voltage activated), Cav2 (P/Q-, N-, and R-type, also high-voltage-activated), and Cav3 (T-type, low-voltage activated). Studies in rodent models have shown that various VGCC subtypes contribute to dendritic Ca^2+^ influx to differing degrees (Markram et al., 1995; Jaafari and Canepari, 2016). In the proximal segments of pyramidal cell dendrites, calcium transients can be elicited through all VGCC subtypes. However, in distal dendritic compartments, where the amplitude of bAP attenuates, Ca^2+^ transients are predominantly mediated by R-type and T-type channels (Christie et al., 1995; Magee and Johnston, 1995; Markram et al., 1995). Consequently, the amplitude of bAP-induced calcium transients is not uniform along the dendritic axis; rather, it forms a spatial profile that varies with dendritic length and differs among various neuronal cell types (Goldberg et al., 2003; Waters et al., 2003; Cho et al., 2010).

To date, much of our understanding of how bAP-mediated dendritic Ca^2+^ signaling supports STDP and other forms of synaptic plasticity comes from studies in model organisms, particularly rodents, with relatively limited research in human neurons and few direct cross-species comparisons. However, as human cortical tissue becomes increasingly available for experimental research, significant differences are being uncovered. Human cortical neurons have larger dendritic and axonal dimensions (DeFelipe, 2011; Mohan et al., 2015; Berg et al., 2021; Oláh et al., 2025) are morphologically more complex and heterogeneous than those of rodents, reflecting cortical expansion during evolution (Gooch et al., 2022; Kanari et al., 2025). These morphological differences contribute to functional distinctions, as dendritic structure profoundly influences the efficacy and speed of action potential (AP) propagation (Vetter et al., 2001; Oláh et al., 2025). Furthermore, human dendrites have unique ion channel compositions (Kalmbach et al., 2018) and a greater number of local dendritic compartments (Eyal et al., 2018; Aizenbud et al., 2024). These integrative differences, combined with distinct synaptic strengths compared to other species, significantly alter input–output relationships and, consequently, cortical computation (Molnár et al., 2008, 2016; Beaulieu-Laroche et al., 2018; Gidon et al., 2020; Hunt et al., 2023; Shapira et al., 2025).

In this study, we performed simultaneous whole-cell patch-clamp recordings and two-photon calcium imaging to investigate bAP-induced Ca^2+^ transients along the apical dendrites of human layer 2/3 (L2/3) cortical pyramidal neurons. We found that back-propagating action potentials in these cells produce a non-monotonic Ca^2+^ signal amplitude profile within the first 150 μm of the apical dendrite, characterized by a proximal peak followed by distal attenuation, similar patterns observed in rodent cortical and hippocampal pyramidal cells. Pharmacological analysis showed that multiple Ca^2+^ channel subtypes contribute to these signals, with N-type channels playing a dominant role in shaping the peak of the profile.

## 2 Materials and methods

### 2.1 Slice preparation

Experiments were performed in accordance with the Declaration of Helsinki and approved by the University of Szeged Ethical Committee. Before surgery, patients provided written consent for all tissue material. We used human cortical tissue that had to be surgically removed from patients (*n* = 14 female, *n* = 20 male) to access deep-brain target malformations (tumors, hydrocephalus, cysts, and aneurysms) for surgical treatment (Supplementary Table 1). Anesthesia was induced with intravenous midazolam (0.03 mg/kg) and fentanyl (1–2 μg/kg), followed by a bolus dose of propofol (1–2 mg/kg) administered intravenously. Patients received 0.5 mg/kg rocuronium to facilitate endotracheal intubation. The trachea was intubated to ventilate the patient with a mixture of O_2_ and N_2_O at a ratio of 1:2. Anesthesia was maintained with sevoflurane at a care volume of 1.2–1.5. During the surgical procedure tissue blocks were removed from various brain regions (parietal: *n* = 5, temporal: *n* = 14, frontal: *n* = 13, occipital: *n* = 2), and the resected tissue blocks were immediately immersed in ice-cold solution. Slices were cut perpendicular to the pia mater at a thickness of 320 μm with a vibrating blade microtome (Microm HM 650 V) in ice-cold solution containing (in mM): 75 sucrose, 84 NaCl, 2.5 KCl, 1 NaH_2_PO_4_, 25 NaHCO_3_, 0.5 CaCl_2_, 4 MgSO_4_, and 25 D(+)-glucose, saturated with 95% O_2_ and 5% CO_2_. The slices were then incubated in the same solution for 30 min at 36 °C. After incubation the solution gradually changed to (in mM): 130 NaCl, 3.5 KCl, 1 NaH_2_PO_4_, 24 NaHCO_3_, 1 CaCl_2_, 3 MgSO_4_ and 10 D(+)-glucose, saturated with 95% O_2_ and 5% CO_2_, and the slices were kept in this solution until use.

### 2.2 *In vitro* electrophysiological recordings

Somatic whole-cell current-clamp recordings were obtained at ~36 °C in artificial cerebrospinal fluid containing (in mM): 130 NaCl, 3.5 KCl, 1 NaH_2_PO_4_, 24 NaHCO_3_, 3 CaCl_2_, 1.5 MgSO_4_ and 10 D(+)-glucose, from layer 2/3 pyramidal cells visualized by infrared differential interference contrast (DIC) video microscopy. Micropipettes (3–5 MΩ) were filled with intracellular solution containing (in mM): 126 potassium-gluconate, 4 KCl, 4 ATP-Mg, 0.3 GTP-Na_2_, 10 HEPES, 10 phosphocreatine, 8 biocytin, 0.015 Alexa hydrazide 594 and 0.1 Oregon Green BAPTA-1 (pH 7.20; 300 mOsm). To investigate Ca^2+^ transients induced by the backpropagation of action potentials to the apical dendrite, one and three action potentials were elicited by brief (5 ms) current pulses injected into the cell body of the neurons.

### 2.3 Two-photon Ca^2+^ imaging

We combined whole-cell recording and Ca^2+^ imaging for the optical recording of dendritic Ca^2+^ signals. The intracellular solution used for the experiments to characterize the Ca^2+^ signals elicited by the backpropagating action potential contained Oregon Green BAPTA-1 (OGB-1, 100 μM) high-affinity Ca^2+^ sensor. After the whole-cell configuration was acquired, the intracellular solution was allowed to diffuse into the cell protrusions for 15–20 min before the two-photon imaging was initiated. Fluorophores were excited by femtosecond Ti:sapphire laser pulses (Mai Tai Deep See; Spectra-physics, United States) at 800 nm wavelength to visualize the dendritic arborization of the pyramidal cells. Intensity changes of the emitted green fluorescence were collected with line scans across the dendrite.

Ca^2+^ signals were analyzed using custom MATLAB (The Math Works, Inc.) scripts. The amplitude of Ca^2+^ transients was calculated as the average of five consecutively recorded Ca^2+^ signals evoked at 2 s intervals and measured at each dendritic region. The Ca^2+^ transients were calculated as the relative change in fluorescence: ΔF/F_0_ = (F − F_0_)/F_0_, where F_0_ is the baseline prestimulus fluorescence, background fluorescence was subtracted from both F and F_0_. Normalized ΔF/F_0_ was calculated by normalizing each Ca^2+^ transient amplitude to the maximum Ca^2+^ signal amplitude measured on the same cell. After the recordings Z-stack images of the cells were acquired, and the distance of the studied dendritic sections from the cell body was measured by tracing the protrusions on the images (ImageJ, Simple Neurite Tracer plugin).

All ion channel blockers were administered extracellularly in the recording solution in our pharmacological experiments. Our experiments included the following pharmacones: SNX-482 (300 nM), NNC 55-0396 dihydrochloride (100 μM), nifedipine (20 μM), ω-conotoxin (1 μM), CdCl_2_ (200 μM), and tetrodotoxin (1 μM).

### 2.4 Histology and reconstruction

Following the two-photon Ca^2+^ imaging experiments, brain slices were prepared for further morphological analysis of the biocytin-labeled cells. Slices were fixed in a fixative solution of 4% paraformaldehyde, 15% picric acid, and 1.25% glutaraldehyde in 0.1 M phosphate (PB, pH = 7.4) for at least 12 h. After multiple washes in 0.1 M PB, slices were immersed in 10% then 20% sucrose solution in 0.1 M PB for cryoprotection. The slices were frozen in liquid nitrogen and then thawed in PB. Slices were embedded in 10% gelatin and sectioned into 70 μm thick sections. Sections were incubated in a solution of conjugated avidin-biotin horseradish peroxidase (ABC; 1:100; Vector Labs) in tris-buffered saline (TBS, pH = 7.4) overnight at 4 °C. The enzyme reaction became visible by using 0.05% 3′3-diaminobenzidine tetrahydrochloride as a chromogen and 0.01% H_2_O_2_ as an oxidant. Sections were post-fixed with 1% OsO_4_ in 0.1 M PB. Following several washes with distilled water, sections were stained in 1% uranyl acetate and dehydrated in an ascending series of ethanol. The sections were infiltrated with epoxy resin (Durcupan, Sigma-Aldrich) overnight and then embedded on glass slides.

Following the visualization of the recorded cells using DAB staining, 3D light microscopic reconstructions were performed using the Neurolucida system (MBF Bioscience, Williston, VT, United States) with a 100× objective. Analysis of morphological features was made by NeuroExplorer software (MBF Bioscience, Williston, VT, United States) and Origin 9 (OriginLab, Northampton, MA). The dendrite diameter was measured at the Ca^2+^ imaging site using 3D reconstructions of the examined cells. Diameters were obtained from intensity profiles using a custom-written script in Igor (Wavemetrics, Lake Oswego, United States) by measuring at 70% of the amplitude (Ozsvár et al., 2018) and were normalized to the diameter at the somatic-dendritic junction. This junction was identified by fitting an ellipsoidal boundary to the soma and selecting the midpoint of the apical stem’s origin. The diameters of the imaged dendritic locations were then averaged within 25 μm dendritic segments starting from the beginning of the apical dendrite. Dendritic spine density was calculated as spines/μm on 25 μm dendrite segments starting from the beginning of the apical dendrite.

### 2.5 Statistics

Data presented as the mean ± S.D. Normality was tested with the Shapiro–Wilk test, for statistical analysis, ANOVA with *post-hoc* Bonferroni test, or Kurskal–Wallis with *post-hoc* Dunn test. For pairwise comparison two-sample *t*-test or Mann–Whitney test was used. Wilcoxon signed ranks or Friedman tests were used for repeated measures. Differences were accepted as significant if *p* < 0.05. The data are shown on boxplots, boxes indicate the 25th, 50th (median), and 75th percentiles, rectangle represents the mean value, and whiskers indicate S.D.

## 3 Results

### 3.1 Amplitude distribution of somatic action potential induced Ca^2+^ transients along the apical dendrite

**Figure 1:**
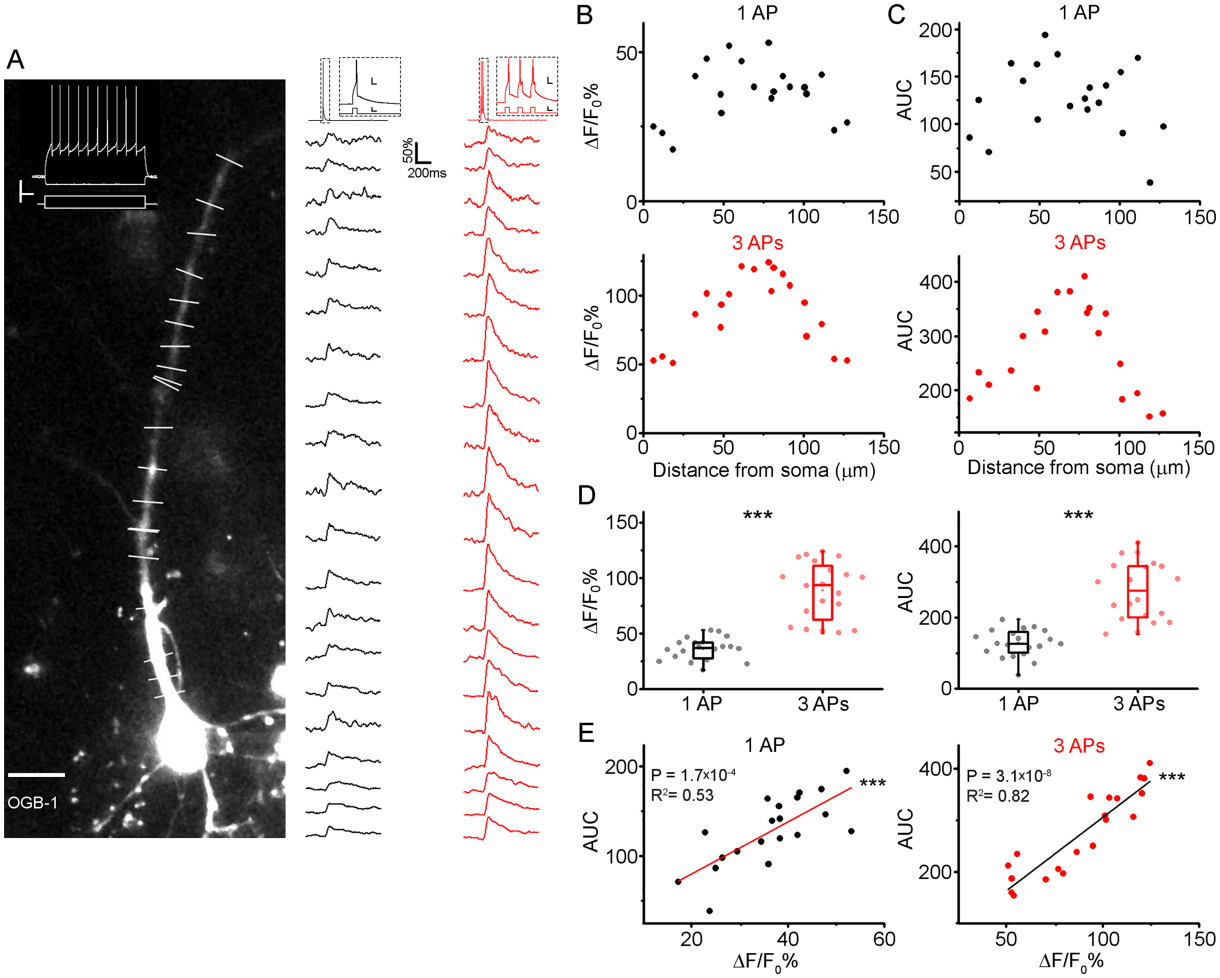
Amplitude distribution of somatic action potential induced Ca^2+^ transients along the apical dendrite. **(A)** Two-photon maximum intensity projection of a representative L2/3 pyramidal cell in the human neocortex filled with OGB-1. Scale for image: 10 μm. Scale for voltage recording and injection current: 20 mV/200 pA/100 ms. Ca^2+^ transients along the apical dendrite evoked by 1 AP (black) or 3 APs (red) from the regions indicated with lines on the left. Inset scales: 20 mV/500 pA/5 ms. **(B)** The distribution of the Ca^2+^ transient amplitudes according to the distance from the soma evoked by one (top, black) or three bAPs (bottom, red). **(C)** Distribution of the area under curve (AUC) of the Ca^2+^ transient according to the distance from the soma evoked by one (top, black) or three bAPs (bottom, red). **(D)** Comparison of all Ca^2+^ signal amplitudes (left, 1 AP: 36.42 ± 9.93%; 3 APs: 88.96 ± 2.6%; *p* = 9.57 × 10^−5^, Wilcoxon signed ranks test) and the area under the curve (right, 1 AP: 128.39 ± 38.8; 3 APs: 275.78 ± 82.14; *p* = 5.23 × 10^−8^, paired sample *t*-test) induced by a single AP or burst. On average, burst-evoked Ca^2+^ responses were approximately twice as large as those induced by a single AP.

Somatically induced action potentials propagate through dendritic branches and activate voltage-gated Ca^2+^ channels throughout the dendrite, causing the influx of the Ca^2+^ into the dendrite. To investigate somatic AP-induced Ca^2+^ signals in the dendrites of human cortical pyramidal cells, we used acutely prepared slices of neurosurgically resected human neocortical tissue. The samples were predominantly obtained from the frontal and temporal lobes of patients who underwent tumor or hydrocephalus surgery. Data were collected from 34 patients aged from 2 to 82 years from cortical layer 2/3 (L2/3) pyramidal cells (*n* = 43, average somatic distance from the *pia mater*: 396.32 ± 88.57 μm). We examined apical and radial oblique dendrites of pyramidal cells using simultaneous somatic whole-cell patch-clamp stimulation and two-photon line scan imaging. Neurons were loaded with the high-affinity Ca^2+^ sensor Oregon Green BAPTA-1 (OGB-1100 μM) via the patch-clamp micropipette (Figure 1A). To characterize the spatial profile of Ca^2+^ transients, we measured the amplitude of Ca^2+^ signals from different locations along the apical dendrite (up to 263 μm from the soma) while evoking single AP or short bursts (three APs) through brief (5 ms) somatic current injections (Figure 1A). The amplitude of Ca^2+^ transients increased with distance from the soma, peaking between 50–100 μm, and subsequently declined in more distal regions. This spatial distribution pattern was consistent regardless of the number of APs elicited (Figure 1B). Ca^2+^ transients elicited by AP bursts were significantly larger at all dendritic locations compared to those evoked by a single backpropagating AP (Figure 1D). To assess whether differences in Ca^2+^ kinetics across dendritic locations might alter the spatial distribution of total Ca^2+^ entry, we also computed the area under the ΔF/F_0_ curve (AUC) for bAP-evoked transients. The AUC exhibited a similar biphasic spatial profile to the peak amplitude, with a maximum in the 50–100 μm segment and reduced values proximally and distally (Figure 1C). Peak amplitude and AUC were highly correlated across all segments (Figure 1E; 1 AP: *R*^2^ = 0.53, *p* = 1.7 × 10^−4^, 3 APs: *R*^2^ = 0.82, *p* = 3.1 × 10^−8^), indicating that local variations in kinetics do not qualitatively change the spatial pattern of bAP-evoked Ca^2+^ influx.

**Figure 2:**
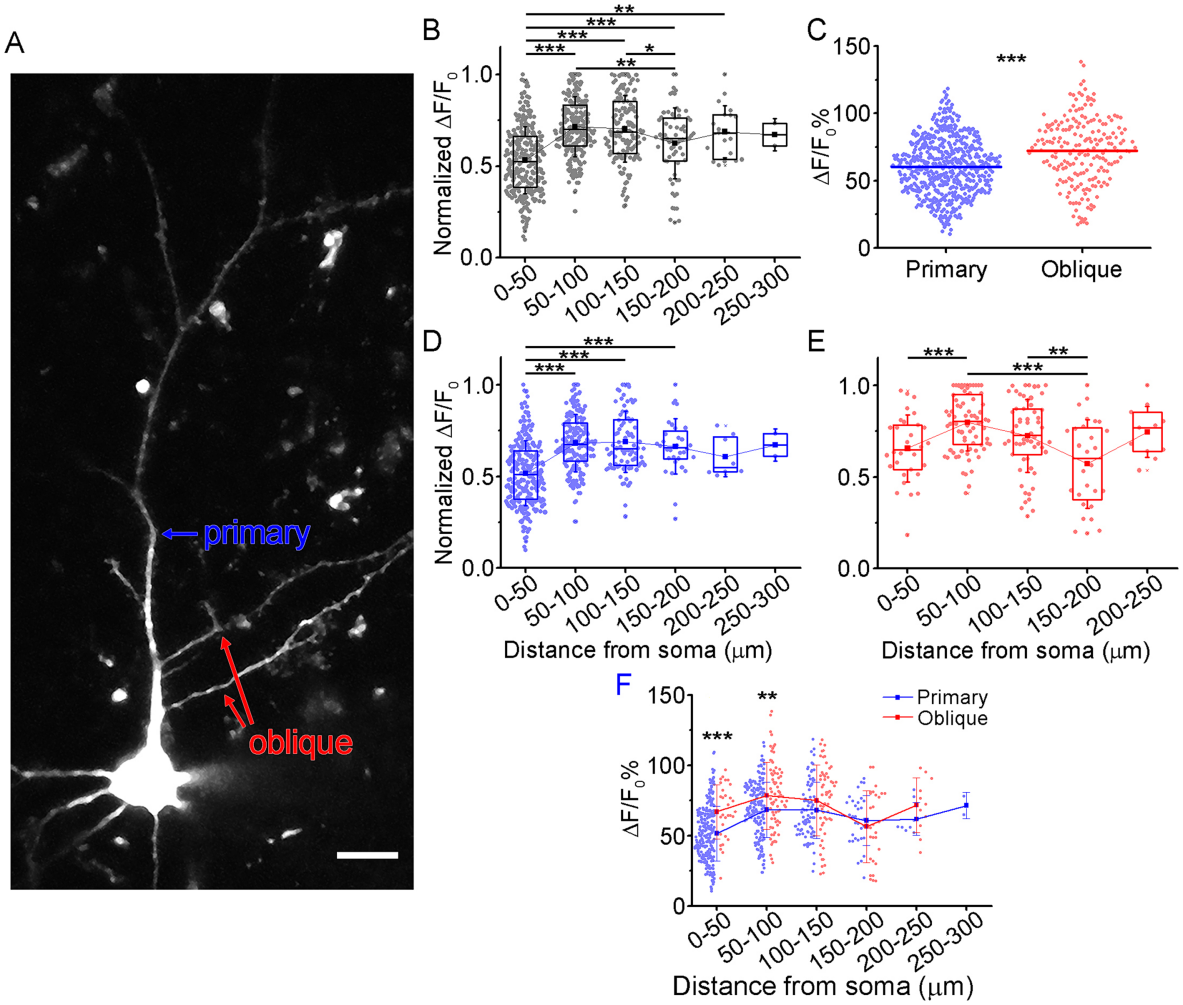
Spatial profiles of Ca^2+^ transient amplitudes differ on the primary and oblique apical dendrites. **(A)** Two-photon maximum intensity projection of a representative L2/3 pyramidal cell. The blue arrow shows the primary apical dendrite, and the red arrows indicate imaged oblique dendrites. Scale: 10 μm **(B)** The normalized spatial profile of all measured Ca^2+^ transients (*n* = 36 cells) of primary and higher order dendrites. The Ca^2+^ transient amplitudes were normalized to the maximum ΔF/F_0_ amplitude in each pyramidal cell. Kruskal–Wallis test: *n* = 272/256/141/66/19/2, *p* = 2.09 × 10^−27^, Dunn *post-hoc* corrected *p*-values: segment1 vs. segment2 *p* = 1.73 × 10^−29^, segment1 vs. segment3 *p* = 2.22 × 10^−16^, segment1 vs. segment4 *p* = 1.84 × 10^−4^, segment1 vs. segment5 *p* = 1.11 × 10^−3^, segment2 vs. segment4 *p* = 2.13 × 10^−3^, segment3 vs. segment4 *p* = 0.02 **(C)** Comparison of Ca^2+^ transient amplitudes on the primary and oblique apical dendrites. Mann–Whitney *U* test, *p* = 3.97 × 10^−10^. The horizontal line indicates the mean of individual Ca^2+^ transients (blue indicates primary and red indicates higher-order dendrites). **(D)** Spatial profile of Ca^2+^ transient amplitudes in primary apical dendrites. Kruskal–Wallis test: *n* = 242/180/83/32/8/2, *p* = 4.22 × 10^−21^, Dunn *post-hoc* corrected *p*-values: segment1 vs. segment2 *p* = 1.73 × 10^−29^, segment1 vs. segment3 *p* = 3.15 × 10^−12^, segment1 vs. segment4 *p* = 1.31 × 10^−5^. **(E)** Same as in **(D)** but measured on radial oblique dendrites. Kruskal–Wallis test: *n* = 30/76/58/28/11, *p* = 6.89 × 10^−5^, Dunn *post-hoc* corrected *p*-values: segment1 vs. segment2 *p* = 6.2 × 10^−4^, segment2 vs. segment4 *p* = 1.35 × 10^−5^, segment3 vs. segment4 *p* = 6.97 × 10^−3^. **(F)** Segment-wise transient amplitude comparison between primary and oblique dendrites along the apical dendrite. The blue (primary) and red (oblique) connected dots represent the mean ΔF/F_0_ amplitude, vertical lines are the S.D., and the dots indicate the individual transient amplitudes. 1st group: Mann–Whitney *U* test, *n* = 242/30, *p* = 1.06 × 10^−4^; 2nd group: two-sample *t*-test, *n* = 180/76, *p* = 1.56 × 10^–3^.

We segmented the imaged dendrites into 50 μm-long sections starting from the soma and computed the average spatial calcium transient amplitudes of *n* = 36 human cortical supragranular pyramidal cells (Figure 2A). We observed a progressive increase in amplitude within the first and second segments followed by a gradual decline in the distal segments after reaching a maximum value (*n* = 756, *p* = 2.09 × 10^−27^, Kruskal–Wallis test) (Figure 2B). To assess differences between dendritic compartments, we compared the magnitudes of Ca^2+^ transients in primary apical dendrites—defined as the thick dendrites emerging directly from the soma—with those in the thinner radial oblique branches. The amplitude of calcium transients was significantly higher in oblique dendrites compared to primary apical dendrites (primary: 60.36 ± 20.98%; oblique: 72.4 ± 24.58%, *n* = 547/203, *p* = 3.97 × 10^−10^, Mann–Whitney *U* test; Figure 2C). The spatial profiles of the calcium transient amplitudes were similar in both primary and oblique dendrites. In both cases, the distribution followed a nonlinear pattern, as described above, with significant variation across segments (primary apical dendrites: *p* = 4.22 × 10^−2^, Kruskal–Wallis test; Figure 2D; oblique dendrites: *p* = 6.89 × 10^−5^, Kruskal–Wallis test; Figure 2E). When comparing the amplitude of transients between the two dendritic types across corresponding segments, significant differences were detected in the first segment (primary: 51.52 ± 19.56%; oblique: 66.92 ± 19.19%, *n* = 242/30, *p* = 1.1 × 10^−4^, Mann–Whitney *U* test,), and in the second segment (primary: 68.46 ± 19.54%; oblique: 78.51 ± 23.88%, *n* = 180/76, *p* = 1.56 × 10^−3^; Figure 2F). No significant differences were observed in the distal dendritic segments.

### 3.2 Correlation between the magnitude of Ca^2+^ transients and morphological characteristics

**Figure 3:**
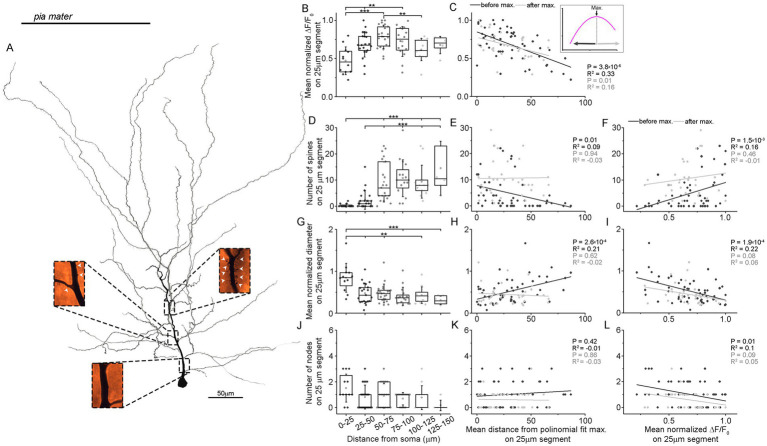
Correlation of Ca^2+^ transients and morphological features. **(A)** Reconstruction of the apical dendrite of a representative layer 2 pyramidal neuron. Inset images show dendritic spine distributions (indicated by white arrowheads) from distinct dendritic regions marked by rectangles. **(B)** Average Ca^2+^ transient distribution for the *n* = 10 reconstructed cells included in the anatomical analysis. **(C)** Calcium signals show a correlation with distance from the soma when measured relative to the location of the maximum signal amplitude, both in the proximal and distal directions on 25 μm long dendritic segments. **(D)** Spine density on imaged primary and oblique apical dendrite segments (*n* = 10, *p* = 9.13 × 10^−9^, Kruskal–Wallis test with *post-hoc* Dunn test). **(E)** The number and location of dendritic spines correlate with the mean normalized Ca^2+^ signal amplitude in 25 μm long dendritic segments from the soma to the peak signal location (*R*^2^ = 0.09, *p* = 0.01), but not in the direction distal to the peak (*R*^2^ = −0.03, *p* = 0.94). Dark and light gray indicate the soma-to-peak and peak-to-distal segments, respectively. **(F)** Spine number as a function of Ca^2+^ signal amplitude demonstrates a strong correlation from the soma to the peak, but not from the peak to distal locations (before peak: *R*^2^ = 0.16, *p* = 1.5 × 10^−3^; after peak: *R*^2^ = −0.01, *p* = 0.46). **(G–I)** Same analyzes as in panels **(D–F)**, but with the correlation of mean normalized dendrite diameter on 25 μm long dendritic segments and the distance from the soma **(G)**, in the correlation between the mean normalized diameter and the distance from the maximal calcium signal (before peak: *R*^2^ = 0.21, *p* = 2.6 × 10^−4^; after peak: *R*^2^ = −0.02, *p* = 0.62) **(H)**, and the mean normalized Ca^2+^ signal amplitude (before peak: *R*^2^ = 0.22, *p* = 1.9 × 10^−4^; after peak: *R*^2^ = 0.06, *p* = 0.08) **(I)** a positive correlation is observed from the soma to the peak signal location, but not beyond. **(J–L)** Same as in **(D–F)**, but with the correlation between the number of bifurcations on 25 μm long dendritic segments and the distance from the soma **(J)**, the mean distance from the maximal signal amplitude **(K)**, and the mean normalized Ca^2+^ signal amplitude **(L)**. No correlation is present with the distance of the maximal signal on the soma to peak segment or from the peak to distally (before peak: *R*^2^ = −0.01, *p* = 0.42; after peak: *R*^2^ = −0.03, *p* = 0.86) **(K)**.

Kinetics and amplitude of intracellular calcium increase evoked by dendritic action potentials are highly dependent on several physical factors, including the distance from the soma, number of branch points, dendritic diameter, input resistance and number of spines. To investigate the influence of dendritic arborization patterns on the spatial distribution of Ca^2+^ transients induced by back-propagating action potentials, we performed three-dimensional reconstructions of the apical dendrites from the examined cells (Figure 3A; Supplementary Figure 1). Our analysis was limited to regions within 150 μm of the soma, and the dendrites were divided into 25 μm-long sections (Figure 3B). We performed correlation analysis on segments located proximal and distal to the location of maximum signal amplitude (Figure 3C). For spine density assessment, we first identified and counted spines on the apical dendrite of *n* = 10 pyramidal cells. The distribution of spine density showed a general increase with distance (average spine number in segments 1–6: 0.33 ± 0.65, 2.43 ± 3.59, 9.28 ± 7.64, 10.75 ± 7.04, 9.38 ± 6.27, 14.5 ± 10.29; *p* = 9.13 × 10^−9^, Kruskal–Wallis test; Figure 3D). In the initial half of the measured apical segment a positive correlation was observed between spine number and the average normalized amplitude of Ca^2+^ signals (before peak: *R*^2^ = 0.09, *p* = 0.01, after peak: *R*^2^ = −0.03, *p* = 0.94; Figures 3E,F). During the three-dimensional reconstructions (*n* = 10) of the biocytin labeled and DAB-stained neurons, we measured dendritic diameter at imaging locations using light microscopy. The normalized diameter showed a gradual decrease with the greatest thickness in the first and second segments (average dendrite diameter in segments 1–6: 2.32 ± 0.94, 1.28 ± 0.66, 1.27 ± 0.61, 1.67 ± 0.56, 1.18 ± 0.65, 1.11 ± 0.49 μm, *p* = 1.12 × 10^−3^, Kruskal–Wallis test; Figure 3G) and was negatively correlated with both the normalized amplitude of Ca^2+^ transients (before peak: *R*^2^ = 0.22, *p* = 1.9 × 10^−4^, after peak: *R*^2^ = 0.08, *p* = 0.06, Figure 3I) and the distance from the soma (before peak: *R*^2^ = −0.02, *p* = 0.21, after peak: *R*^2^ = 0.62, *p* = 2.6 × 10^−4^; Figure 3H). Additionally, the number of dendritic nodes quantified from the reconstructions (*n* = 10) showed no correlation with increasing distance (average number of nodes in segments 1–6: 1.5 ± 1.09, 0.81 ± 0.93, 0.9 ± 0.94, 0.69 ± 0.48, 0.77 ± 0.93, 0.17 ± 0.41, *p* = 0.09, Kruskal–Wallis test; Figure 3J), and a slight correlation was found between node number and the normalized amplitude of Ca^2+^ transients (before peak: *R*^2^ = 0.1, *p* = 0.01, after node: *R*^2^ = 0.05, *p* = 0.09; Figures 3K,L). Thus, the amplitude profile of the Ca^2+^ signal induced by bAP appears to correlate to some extent with dendritic geometry, specifically, spine density correlated positively, while diameter correlated negatively with the rise profile of the proximal calcium signal.

### 3.3 Contribution of Ca^2+^ channel subtypes to the amplitude of Ca^2+^ signals in the apical dendrite

**Figure 4:**
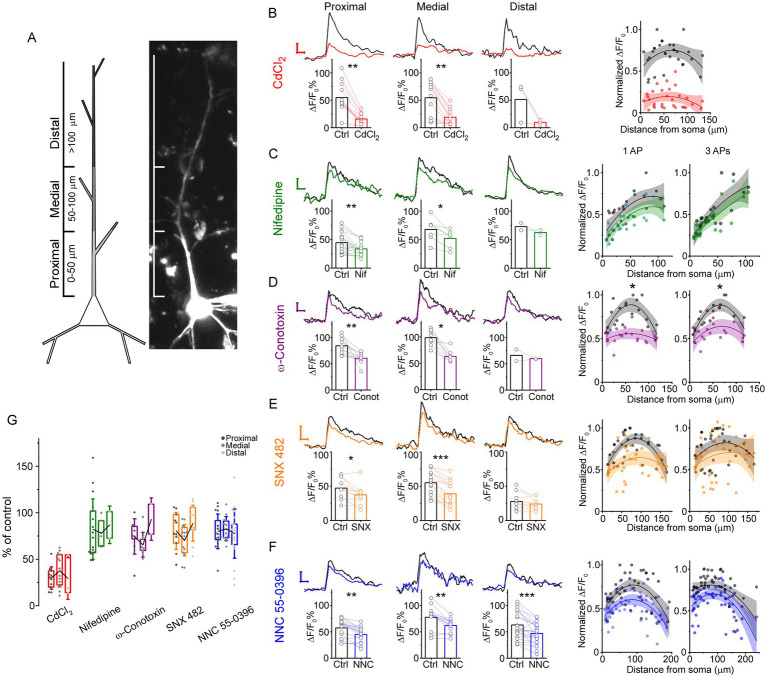
Effect of specific VGCC inhibitors on Ca^2+^ signals in different dendritic regions. **(A)** Left: Schematic illustration of pyramidal neuron segmentation. Right: Two-photon image of a human L2/3 pyramidal neuron indicating the proximal, medial, and distal dendritic regions. **(B)** Ca^2+^ transients evoked by backpropagating AP before and after application of CdCl_2_ (200 μM). Representative traces from the proximal, medial, and distal dendritic regions are shown above: black traces represent control conditions, and red traces represent signals after inhibition of VGCC. Scale bars: *x* = 100 ms, *y* = 20%. Bar graphs display the average Ca^2+^ signal amplitudes, with paired data points representing measurements from the same imaging site before and after drug application across dendritic regions (*n* = 4 cells, proximal: *n* = 13 imaging sites, *p* = 1.66 × 10^−3^; medial: *n* = 13 imaging sites, *p* = 1.66 × 10^−3^; distal: *n* = 3 imaging sites, *p* = 0.18, Wilcoxon signed ranks test). Right: Scatter plot of Ca^2+^ signal amplitude versus distance from the soma, with second-order polynomial fits applied to control and drug-treated conditions (control: *n* = 30, *R*^2^ = 0.08, *p* = 0.12, CdCl_2_: *n* = 30, *R*^2^ = 0.01, *p* = 0.31). The shaded areas indicate the 95% confidence interval. All Ca^2+^ responses were elicited by single AP stimulation. **(C–F)** Same experimental design as in panel **(B)**, using specific VGCC inhibitors nifedipine **(C)** (*n* = 5 cells, proximal: *n* = 17 imaging sites, *p* = 5.84 × 10^−3^, paired sample *t*-test; medial: *n* = 7 imaging sites, *p* = 0.022; distal: *n* = 2 imaging site, *p* = 1, Wilcoxon signed ranks test), ω-conotoxin **(D)** (*n* = 2 cells, proximal: *n* = 11 imaging sites, *p* = 8.05 × 10^−3^; medial: *n* = 9 imaging sites, *p* = 0.01; distal: *n* = 2 imaging sites, *p* = 1, Wilcoxon signed ranks test), SNX-482 **(E)** (*n* = 3 cells, proximal: *n* = 11 imaging sites, *p* = 0.01; medial: *n* = 16 imaging sites, *p* = 2.8 × 10^−6^; distal: *n* = 10 imaging site, *p* = 0.15, Wilcoxon signed ranks test), and NNC 55-0396 **(F)** (*n* = 4 cells, proximal: *n* = 17 imaging sites, *p* = 1.78 × 10^−3^; medial: *n* = 13 imaging sites, *p* = 3.3 × 10^−3^, Wilcoxon signed ranks test; distal: *n* = 21 imaging site, *p* = 8.79 × 10^−4^, paired sample *t*-test). The scale bars for Ca^2+^ transients are consistent with panel **(B)**. Right: Corresponding scatter plots show amplitude changes across dendritic locations, with responses elicited by either a single AP (1 AP) or a brief burst (3 AP). Significant reductions in Ca^2+^ signal amplitude were observed in the proximal and medial segments for nifedipine, ω-conotoxin, and SNX-482, and in all segments (proximal, medial and distal) for NNC 55-0396. **(G)** Summary of percent change in Ca^2+^ signal amplitude relative to control for each VGCC blocker in each dendritic segment.

To clarify the contribution of different Ca^2+^ channel subtypes to Ca^2+^ signaling in apical dendrites, we performed experiments using VGCC blockers. First, we used the nonspecific calcium channel blocker CdCl_2_ (200 μM, *n* = 4) and observed a significant reduction in the amplitude of Ca^2+^ transients compared to control at the same dendritic sites (control: 55.25 ± 28.18% CdCl_2_: 16.69 ± 11.53%, *p* = 1.83 × 10^−6^, Wilcoxon signed ranks test). We then divided the Ca^2+^ signals into three 50 μm-long segments relative to the soma, 0–50 μm (proximal), 50–100 μm (medial) and 100–150 μm (distal) along the apical dendrite (Figure 4A). Nonspecific blockade of VGCCs resulted in a significant decrease in Ca^2+^ signal amplitude in the proximal (control: 54.95 ± 25.72%, CdCl_2_: 15.88 ± 9.51%; *p* = 1.66 × 10^−3^, Wilcoxon signed ranks test) and medial segments (control: 54.56 ± 31.3%, CdCl_2_: 19.02 ± 14.43%; *p* = 1.66 × 10^−3^, Wilcoxon signed ranks test) of the apical dendrite (Figure 4B). To evaluate changes in the different segments, we performed curve fitting on the calcium signal profiles. The distribution of Ca^2+^ transient amplitude was best fit with a second-order polynomial function (Figure 4B). We compared the quadratic (a) and linear (b) coefficients of the fits and found no significant difference in the slope (*p* = 0.312) and linearity (*p* = 0.316) coefficients.

Next, we focused on high-voltage-activated L-, N- and R-type calcium channels. The L-type VGCC antagonist nifedipine (20 μM, *n* = 5), the N-type VGCC antagonist ω-conotoxin (1 μM, *n* = 2) and the R-type channel antagonist SNX 482 (300 nM, *n* = 3) significantly decreased the amplitudes of Ca^2+^ signals (nifedipine: *n* = 26, *p* = 5.52 × 10^−4^; ω-conotoxin: *n* = 21, *p* = 1.75 × 10^−4^; SNX 482: *n* = 37, *p* = 1.49 × 10^−6^, Wilcoxon signed ranks test). Nifedipine reduced the amplitude of Ca^2+^ transients in dendritic segments located within 100 μm of the cell body (proximal: control: 44.57 ± 18.28%, nifedipine: 33.72 ± 10.7%, *n* = 17, *p* = 5.84 × 10^−3^, paired sample *t*-test) and in the medial segment (control: 67.62 ± 20.08%, nifedipine: 52.25 ± 15.32%, *n* = 7, *p* = 0.022, Wilcoxon signed ranks test; Figure 4C). Similarly, treatment with ω-conotoxin led to a decrease in the amplitude of fluorescence transients in both the proximal (control: 83.89 ± 15.54%, ω-conotoxin: 60.27 ± 11.04%, *n* = 11, *p* = 8.05 × 10^−3^, Wilcoxon signed ranks test) and medial (control: 98.56 ± 15.02%, ω-conotoxin: 62.96 ± 11.4%, *n* = 9, *p* = 0.01, Wilcoxon signed ranks test) regions of the apical dendrite (Figure 4D). Blocking R-type VGCCs by bath-applied SNX 482 resulted in a significant decrease in Ca^2+^ signals in the proximal (control: 47.22 ± 14.56%, SNX 482: 37.44 ± 14.05%, *n* = 11, *p* = 0.01, Wilcoxon signed ranks test) and medial segments (control: 55.14 ± 17.59%, SNX 482: 39.16 ± 17.37%, *n* = 16, *p* = 2.8 × 10^−6^, Wilcoxon signed ranks test), but not in the distal regions (control: 27.87 ± 12.44%, SNX 482: 24.02 ± 8.03%, *n* = 10, *p* = 0.15, Wilcoxon signed ranks test; Figure 4E). Comparison of curve fits of amplitude profiles revealed no significant differences between L- and R-type channel blocking, with no significant differences in slope (*p* = 0.835 and 0.132 for nifedipine and SNX 482, respectively) and linearity coefficients (*p* = 0.86 and 0.09 for nifedipine and SNX 482, respectively). However, for ω-conotoxin, the slope and linearity of the fitted curve were significantly different (*p* = 0.015, *p* = 0.014).

To test the contribution of the low-voltage-activated calcium channel we applied the T-type Ca^2+^ channel inhibitor NNC 55-0396 (100 μM, *n* = 4), which resulted in a significant decrease in Ca^2+^ signals (*n* = 51, *p* = 5.29 × 10^−8^, Wilcoxon signed ranks test). In different dendritic regions we also found in all regions tested (proximal: control: 57.43 ± 17.38%, NNC 55-0396: 44.94 ± 14.46%, *n* = 17, *p* = 1.78 × 10^−3^, Wilcoxon signed ranks test; medial: control: 77.74 ± 22.27%, NNC 55-0396: 62.28 ± 13%, *n* = 13, *p* = 3.3 × 10^−3^, Wilcoxon signed ranks test; distal: control: 63.13 ± 25.84%, NNC 55-0396: 47.21 ± 19.83%, *n* = 21, *p* = 8.79 × 10^−4^, paired sample *t*-test; Figure 4F). We found no significant change in the fitted curve coefficients representing initial slope (*p* = 0.392) and linearity (*p* = 0.415) (Figure 4F).

In summary, we found that cadmium significantly reduced but did not completely eliminate the voltage-activated calcium transients. By selectively blocking L-, N-, R-, and T-type calcium channels, we found that each contributed to the intracellular Ca^2+^ signaling of bAP in similar proportions overall. When the apical dendrites were divided into segments, the N-type calcium channel caused the greatest change in calcium signaling in the examined middle part of the dendrite segment (Figure 4G).

## 4 Discussion

In this study, we recorded Ca^2+^ signals in the apical dendrites of human cortical supragranular pyramidal neurons during single action potentials or brief bursts providing the first high-resolution spatial map of Ca^2+^ signals. Within the initial 150 μm of the apical trunk and its oblique (radial) dendrites, our data reveal a biphasic spatial profile of bAP-evoked Ca^2+^ transients: signal amplitudes progressively increase with distance from the soma, peak within the dendritic segment located 50–100 μm from the soma, and then decrease in more distal regions. This amplitude distribution profile does not correlate with the number of branch points; instead, within the proximal half of the investigated dendritic section we observed correlations with dendritic diameter and spine number. Somatically evoked dendritic Ca^2+^ influx arises from the synergistic activation of high-voltage-activated and low-voltage-activated Ca^2+^ channels, including L-, N-, R-, and T-type channels. Among these, N-type channels showed the most significant impact on the shape of the spatial amplitude profile, particularly in the medial (50–100 μm) segment.

A number of studies have explored propagation of somatic action potentials and the resulting Ca^2+^ conductance in apical dendrites, and have consistently reported a biphasic spatial development of Ca^2+^ signals in cortical and hippocampal pyramidal cells of various rodent species (Regehr et al., 1989; Jaffe et al., 1992; Miyakawa et al., 1992; Schiller et al., 1995; Svoboda et al., 1997, 1999; Waters et al., 2003; Frick et al., 2004). In these rodent studies, the peak of the Ca^2+^ profile typically occurs around 100 μm from the soma, which is strikingly similar to the profile we observe in human L2/3 cells, despite numerous species-specific differences. Human pyramidal neurons differ fundamentally from rodent neurons in their overall morphological extent and apical dendrite thickness, as well as in their signal propagation (Mohan et al., 2015; Gooch et al., 2022; Oláh et al., 2025). Human cells also appear to possess greater dendritic computational capacity (Shapira et al., 2025), and the time-dependent plasticity of spines triggered on the apical trunk differ significantly from that typically found in rodent brains (Verhoog et al., 2013). The similarity in the extent and kinetics of the Ca^2+^ profile between species likely reflects a conserved interplay between basic morphological parameters and ion channel distributions.

It is important to note that our measurements do not directly resolve the membrane voltage waveform along the apical dendrite, so we cannot fully separate the contributions of bAP voltage propagation from those of Ca^2+^ channel activation. However, studies in rodent pyramidal neurons indicate that the fast Na^+^ component of the bAP generally attenuates monotonically with distance along the apical trunk (Stuart and Sakmann, 1994; Spruston et al., 1995; Stuart et al., 1997b; Harnett et al., 2013). In this context, the biphasic profile of bAP-evoked Ca^2+^ signals we observe in human L2/3 neurons is most parsimoniously explained by distance-dependent changes in surface-to-volume ratio, dendritic morphology, and Ca^2+^ channel composition and gating, rather than by a strongly non-monotonic voltage profile.

The initial increasing phase of the profile is likely driven by several factors, including changes in the surface-to-volume ratio of the dendrite as it tapers and the density or properties of endogenous Ca^2+^ buffers. The subsequent decline in distal regions can be attributed to a combination of factors: attenuation of the propagating Na^+^ action potential with distance from the soma (Stuart and Sakmann, 1994; Spruston et al., 1995; Stuart et al., 1997a; Gidon et al., 2020) and increased K^+^ channel density that reduces bAP amplitude in distal dendrites (Gasparini et al., 2007; Harnett et al., 2013). In addition, voltage-gated Na^+^ channels density decreases with distance from the soma, further contributing to bAPs attenuation (Lorincz and Nusser, 2010). The composition and likely the density of Ca^2+^ channels also change with distance: a proximal predominance of HVA VGCCs gives way to relatively greater LVA VGCC contributions in more distal regions (Christie et al., 1995) which may further shape the transition from peak segment to distal attenuation. Thus, our data emphasize that dendritic Ca^2+^ signals represent a nonlinear, compartment-specific transformation of membrane voltage, and should not be interpreted as a simple linear proxy for bAP amplitude.

We observed significantly higher Ca^2+^ signal amplitude in oblique dendrites compared to the primary apical trunk, despite broadly similar spatial profiles along each branch type. This difference likely reflects a combination of factors, including differences of dendritic diameter and hence surface-to-volume ratio (Schiller et al., 1995), branch-specific distribution of dendritic K^+^ channels (Hoffman et al., 1997; Frick et al., 2004) and potential differences in VGCC density between branch types (Landau et al., 2022). Higher Ca^2+^ conductance in oblique dendrites supports the view that these branches function as distinct biochemical and computational compartments, potentially serving as hotspots for local plasticity (LTP/LTD) that can be partially independent of the main trunk.

Our pharmacological data confirm that dendritic VGCCs are the primary mediators of Ca^2+^ transients in human supragranular neurons. We observed contributions from both LVA and HVA Ca^2+^ channels. Specifically, R-, L-, and N-type channels significantly affected Ca^2+^ signals in proximal (<50 μm) and medial (50–100 μm) segments, whereas T-type channels contributed across all segments examined. Our data suggest that as the bAP traverses the peak region (50–100 μm) LVA T-type channels may provide a substantial source of Ca^2+^ influx in more distal regions, thereby maintaining a window for synaptic integration even when the bAP is strongly attenuated (Markram and Sakmann, 1994; Christie et al., 1995; Magee and Johnston, 1995; Basak and Narayanan, 2018). Notably, ω-conotoxin was the only blocker that significantly altered both the linear and quadratic components (slope and curvature) of the spatial amplitude profile. This finding is consistent with a non-uniform distribution of N-type channels. While nonspecific blockade with CdCl_2_ mostly scaled down the profile without substantially changing its shape, N-type channel blockade altered the spatial pattern itself, suggesting that N-type channels may be selectively enriched within the 50–100 μm peak zone and contribute critically to the characteristic nonlinear amplification in this region. As a limitation, pharmacological separation of individual current components from a mixed population of structurally related channels inevitably suffers from imperfect selectivity (Grantham et al., 1994) and incomplete block of some channel types. Another limitation of our work is that in some segments profiling with blockers is based on a small number of cells. Future progress will likely require combinatorial genetic–pharmacological strategies, higher-resolution imaging, and detailed anatomical mapping, particularly extending to distal tufts in the human cortex.

Although our pharmacological profiling identifies contributions from L-, N-, R- and T-type channels to bAP-evoked Ca^2+^ signals in human supragranular dendrites, it is important to emphasize that detailed subcellular maps of VGCC subtype distribution in human pyramidal neurons are not yet available. Most knowledge about the spatial organization of LVA and HVA channels along dendrites and spines comes from rodent preparations, where two-photon Ca^2+^ imaging, EM and subtype-selective blockers have been combined to show prominent dendritic and spine expression of L-, R- and T-type channels. In human cortex, direct evidence has so far been largely restricted to somatic recordings and bath application of L-type blockers, which demonstrate a critical role for L-type channels in spike-timing-dependent plasticity (Verhoog et al., 2013) but do not resolve their precise dendritic localization or the relative contribution of other subtypes. Our interpretation that R- and T-type channels support bAP-evoked dendritic Ca^2+^ in humans therefore relies on the convergence between our pharmacological effects and these rodent data, together with the conserved expression of VGCC gene families across species. Accordingly, we view the present results as a first *in situ* demonstration that multiple VGCC subtypes shape bAP-evoked Ca^2+^ profiles in human apical dendrites, while acknowledging that definitive subtype-specific distributions and quantitative contributions at the level of individual branches remain to be determined.

The nonlinear spatial profile of bAP-evoked Ca^2+^ signals appears to be a general property of apical dendrites across pyramidal neuron types. Furthermore, Ca^2+^ influx can vary substantially between individual dendritic branches independently of their distance from the soma. In layer 5 pyramidal neurons, for instance, primary apical dendrites and oblique branches exhibit distinct Ca^2+^ signal amplitudes in response to bAPs, probably due to differential ion channel expression and local membrane properties. Dendritic morphology strongly influences the amplitude and kinetics of Ca^2+^ transients. Dendritic diameter and surface-to-volume ratio critically affect Ca^2+^ dynamics (Holthoff et al., 2002; Crandall et al., 2010), with thinner, more distal dendrites often exhibiting faster kinetics and smaller amplitudes due to their geometry (Anwar et al., 2014; Hayward and Cohen, 2025). Increased branching complexity lowers local input resistance and attenuates bAPs, thereby diminishing associated Ca^2+^ signals (Spruston et al., 1995; Williams and Stuart, 2000; Vetter et al., 2001; Frick et al., 2003; Cai et al., 2004; Gasparini et al., 2007; Harnett et al., 2013). The modest correlation we observed between spine density and Ca^2+^ transient amplitude may reflect underlying associations with dendritic diameter and distance from the soma. Spines can act as local sinks for Ca^2+^, but a high density of voltage-gated channels on spines or at spine necks could also contribute to the global dendritic signal (Holthoff et al., 2002; Landau et al., 2022). The correlation suggests that spine-rich regions are either more excitable or host higher Ca^2+^ buffering capacities that are transiently overwhelmed during APs. Spine density has been associated with the speed of bAP propagation in dendrites of similar diameter (Tian et al., 2022), and variations in spine morphology—such as spine neck resistance and head volume—can differentially affect bAP invasion and local Ca^2+^ influx (Trong et al., 2017). bAP invade dendritic spines and can evoke larger Ca^2+^ transients in spines than in adjacent shafts (Yuste and Denk, 1995; Majewska et al., 2000), further contributing to spatially heterogeneous Ca^2+^ signaling.

The density and distribution of inhibitory synapses also shape bAP-evoked Ca^2+^ signals. GABAergic inhibition can dampen dendritic excitability; for example, blockade of GABA_A_ receptors increases the amplitude of distal Ca^2+^ transients (Larkum et al., 2007). In human cortex, dendritic signal processing is further complicated by human-specific interneuron types. Segment-specific suppression of bAP-evoked Ca^2+^ signals in apical dendrites has been attributed to inhibitory input from rosehip cells (Boldog et al., 2018). Such pronounced nonlinearity and compartmentalization are thought to enhance the storage and processing capabilities of neurons and circuits (Poirazi et al., 2003; Aizenbud et al., 2024; Shapira et al., 2025).

Despite the many morphological and functional differences between human and rodent pyramidal cells, our study indicates that the basic spatial distribution of action potential-evoked Ca^2+^ signals near the soma is preserved. This conserved pattern likely places important constraints on how information is integrated and stored in pyramidal neurons across species, while still allowing for species-specific specializations in distal dendrites and branch-specific computation.

## Data Availability

The original contributions presented in the study are included in the article/Supplementary material, further inquiries can be directed to the corresponding author.

## Supplementary material

SUPPLEMENTARY FIGURE 1 Reconstructions of the apical dendrites of human cortical L2 pyramidal cells.

SUPPLEMENTARY TABLE 1 Details showing the patient data used in the experiments of this study.
