# Supplementary figures and images for: Spatial characterization of backpropagating action potential-evoked Ca2+ signals in human cortical layer 2/3 pyramidal neurons
Source: Front Synaptic Neurosci. 2026 Feb 10;18:1769881. doi: 10.3389/fnsyn.2026.1769881 (PMC12929537; doi:10.3389/fnsyn.2026.1769881)

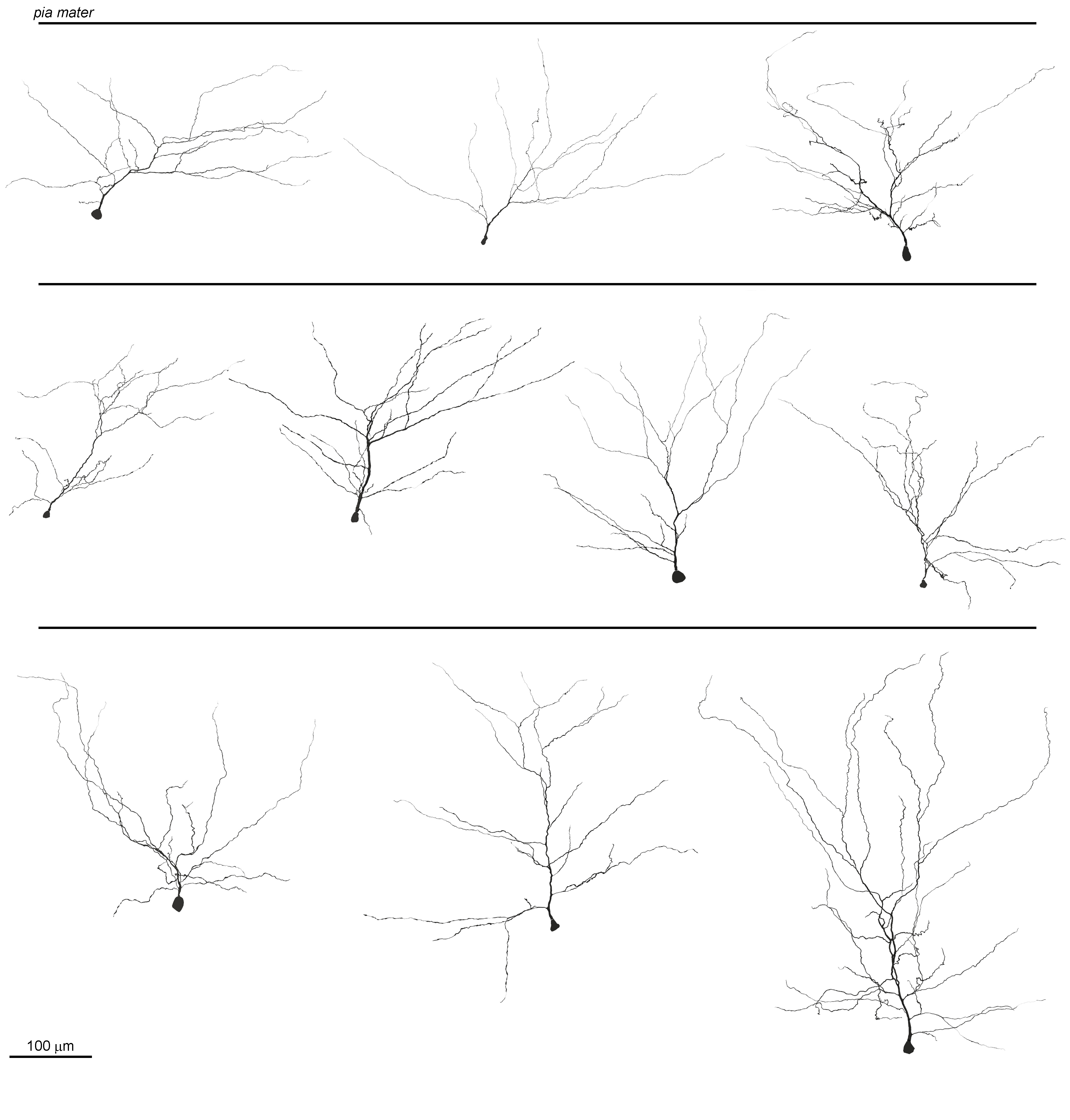

Supplement: SUPPLEMENTARY FIGURE 1 — Reconstructions of the apical dendrites of human cortical L2 pyramidal cells. [file Image_1.JPEG]
