# Supplementary material for: Spatial characterization of backpropagating action potential-evoked Ca2+ signals in human cortical layer 2/3 pyramidal neurons
Source: Front Synaptic Neurosci. 2026 Feb 10;18:1769881. doi: 10.3389/fnsyn.2026.1769881 (PMC12929537; doi:10.3389/fnsyn.2026.1769881)
Supplement: SUPPLEMENTARY TABLE 1 — Details showing the patient data used in the experiments of this study. [file Table_1.DOCX]

**Supplementary Table 1**

| **Age (years)** | **Sex** | **Brain region** | **Hemisphere** | **Medical condition** | **Number of cells utilized** |
| --- | --- | --- | --- | --- | --- |
| 74 | Female | Frontal | Right | Tumor | 2 |
| 43 | Male | Temporal | Right | Tumor | 1 |
| 4 | Male | Temporal | Left | Tumor | 2 |
| 2 | Male | Frontal | Left | Hydrocephalus | 3 |
| 21 | Female | Frontal | Right | Hydrocephalus | 2 |
| 37 | Female | Temporal | Right | Hydrocephalus | 1 |
| 76 | Male | Parietal | Left | Tumor | 1 |
| 68 | Male | Parietal | Right | Tumor | 1 |
| 49 | Female | Temporal | Left | Tumor | 1 |
| 69 | Female | Temporal | Right | Tumor | 2 |
| 72 | Female | Temporal | Right | Tumor | 1 |
| 70 | Male | Temporal | Right | Tumor | 1 |
| 72 | Male | Temporal | Right | Hydrocephalus | 1 |
| 68 | Male | Frontal | Left | Tumor | 1 |
| 34 | Female | Temporal | Right | Tumor | 2 |
| 74 | Female | Temporal | Right | Hydrocephalus | 1 |
| 65 | Female | Frontal | Right | Colloid cyst | 1 |
| 65 | Female | Parietal | Right | Aneurysm | 1 |
| 45 | Male | Frontal | Right | Tumor | 1 |
| 75 | Male | Occipital | Left | Tumor | 1 |
| 44 | Male | Frontal | Left | Tumor | 2 |
| 41 | Male | Temporal | Left | Traumatic haemorrhage | 1 |
| 43 | Female | Frontal | Right | Hydrocephalus | 1 |
| 82 | Female | Temporal | Left | Tumor | 1 |
| 68 | Male | Parietal | Right | Hydrocephalus | 1 |
| 53 | Female | Frontal | Left | Tumor | 2 |
| 57 | Male | Frontal | Right | Hydrocephalus | 1 |
| 77 | Male | Occipital | Left | Hydrocephalus | 1 |
| 66 | Male | Frontal | Right | Hydrocephalus | 1 |
| 43 | Male | Frontal | Right | Hydrocephalus | 1 |
| 67 | Male | Temporal | Right | Tumor | 1 |
| 64 | Female | Temporal | Right | Hydrocephalus | 1 |
| 69 | Male | Parietal | Right | Hydrocephalus | 1 |
| 58 | Male | Frontal | Right | Tumor | 1 |
